# Modeling and Simulation of Process Technology for Nanoparticulate Drug Formulations—A Particle Technology Perspective

**DOI:** 10.3390/pharmaceutics13010022

**Published:** 2020-12-24

**Authors:** Jens Uhlemann, Holger Diedam, Werner Hoheisel, Tobias Schikarski, Wolfgang Peukert

**Affiliations:** 1 Bayer SAS, Environmental Science, 16, Rue Jean-Marie Leclair, 69266 Lyon CEDEX 09, France; 2 Bayer AG, Engineering & Technology, Applied Mathematics, Building B106, 102, 51368 Leverkusen, Germany; holger.diedam@bayer.com; 3 Invite GmbH, Formulation Technology, Building W 32, 51368 Leverkusen, Germany; hoheisel@invite-research.com; 4 Department Chemical and Biochemical Engineering (CBI), Institute of Particle Technology (LFG) and Center for Functional Particle Systems, Cauerstraße 4, 91058 Erlangen, Germany; tobias.schikarski@fau.de

**Keywords:** nanocrystal, poorly soluble drug, precipitation, comminution, oral bioavailability, modeling and simulation, product design, pharmaceutical material science

## Abstract

Crystalline organic nanoparticles and their amorphous equivalents (ONP) have the potential to become a next-generation formulation technology for dissolution-rate limited biopharmaceutical classification system (BCS) class IIa molecules if the following requisites are met: (i) a quantitative understanding of the bioavailability enhancement benefit versus established formulation technologies and a reliable track record of successful case studies are available; (ii) efficient experimentation workflows with a minimum amount of active ingredient and a high degree of digitalization via, e.g., automation and computer-based experimentation planning are implemented; (iii) the scalability of the nanoparticle-based oral delivery formulation technology from the lab to manufacturing is ensured. Modeling and simulation approaches informed by the pharmaceutical material science paradigm can help to meet these requisites, especially if the entire value chain from formulation to oral delivery is covered. Any comprehensive digitalization of drug formulation requires combining pharmaceutical materials science with the adequate formulation and process technologies on the one hand and quantitative pharmacokinetics and drug administration dynamics in the human body on the other hand. Models for the technical realization of the drug production and the distribution of the pharmaceutical compound in the human body are coupled via the central objective, namely bioavailability. The underlying challenges can only be addressed by hierarchical approaches for property and process design. The tools for multiscale modeling of the here-considered particle processes (e.g., by coupled computational fluid dynamics, population balance models, Noyes–Whitney dissolution kinetics) and physiologically based absorption modeling are available. Significant advances are being made in enhancing the bioavailability of hydrophobic compounds by applying innovative solutions. As examples, the predictive modeling of anti-solvent precipitation is presented, and options for the model development of comminution processes are discussed.

## 1. Introduction

Modeling and simulation of the full oral delivery process chain for drug formulations can serve the ultimate task of accurately predicting in vivo pharmacokinetics of a new potential drug [1] by providing a quantitative model for drug manufacturing and delivery. This is of particular interest for the many new molecular entities identified by pharmaceutical industry screening programs exhibiting poor water solubility [2], which makes their formulation difficult or even impossible. Applying a range of nano-based solutions to improve the drug dissolution and bioavailability of hydrophobic compounds is a promising approach if specific conditions for the drug delivery challenges are met. For the purpose of this article, organic drug nanoparticles (ONP) are defined as solid organic particles with a mean diameter <1 μm having either a crystalline or amorphous character. Liposomes and micelles as drug nanocarriers are here excluded from the terminology nanoparticle.

**Figure 1:**
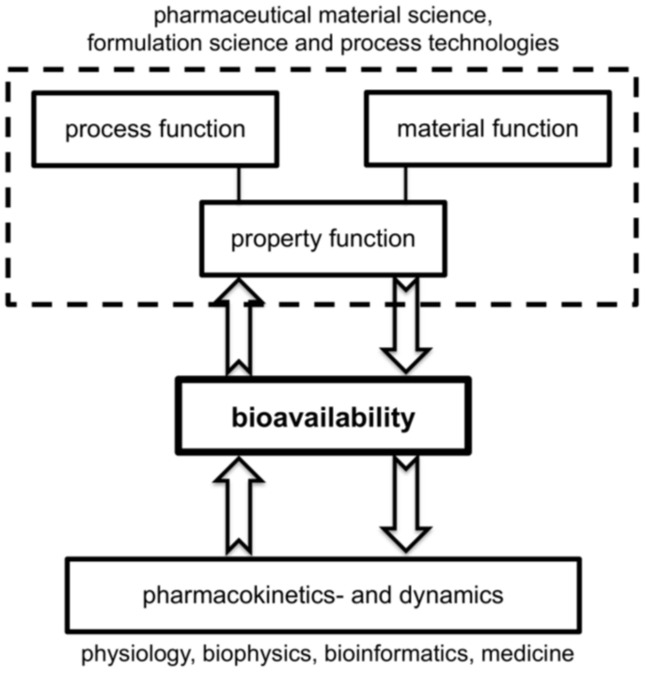
Long-term objective for quantitative prediction of bioavailability via the interplay of models for process technologies of active pharmaceutical ingredients (API) production and whole-body pharmacological modeling.

Any comprehensive digitalization of drug formulation requires the combination of pharmaceutical material and formulation science coupled to adequate process technologies on the one hand and quantitative pharmacokinetics and dynamics of drug administration in the human body on the other side. The combination of process and material models in a formulation process leads to the desired property function. Both approaches use process models for the technical realization of the drug production and the distribution of the pharmaceutical compound in the human body and are closely coupled via the central objective, namely bioavailability; see Figure 1.

The process models for the property function can complement models for bioavailability based on quantitative structure–property relationship (QSPR), physiologically based–pharmacokinetic (PBPK) and rule-of-thumb (RoT) approaches [1,3,4]. These approaches are currently being utilized independently. Future developments of promising tools could be based on combining these process models with hybrid QSPR-PBPK approaches together with the exploration of ensemble and deep-learning systems for QSPR modeling.

Sole machine learning or artificial intelligence-based algorithms are used in many applications along the pharmaceutical development pipeline [5], whereas the prediction of physical chemical properties of compounds such as distribution equilibria, solubility, or melting point [6], as well as more complex tasks, for instance absorption, distribution, metabolism, excretion, and toxicity [7] or retrosynthesis [8], the in silico prediction of formulation performance is far less established. Nevertheless, such data-driven models can be utilized to support formulation development in a wide range of different quantities, e.g., in vitro performance [9], stability [10], or disintegration time [11]. In particular, for nanoformulations, the published data-driven methods range from a linear approach [12] to a recent publication [13] that developed multiple machine learning models for the prediction of the nanocrystal size and polydispersity index (PDI) for multiple different manufacturing methods.

The objective of the current paper is to discuss the combination of absorption, distribution, metabolism, and excretion (ADME) modeling with the modeling of ONP manufacturing processes. Digital pharmaceutics entails modeling of the manufacturing process, e.g., the precipitation of ONP with a particle technology-based approach, as well as modeling of gastro-intestinal transit and absorption by a physiologically-based pharmacokinetic (PBPK) model for gastro-intestinal transit and absorption combined with a mechanistic dissolution model of the Noyes–Whitney type. The entire modeling approach is based on strategies established in product design and engineering, digital pharmaceutics, as well as pharmaceutical materials science.

The outline of the paper is as follows. Section 2 discusses the state-of-the-art in poorly soluble drug formulation routes to further clarify the requirements and the application scope for nanoparticle-based oral delivery. Critical hurdles and objectives for drug formulation are discussed. The application of the pharmaceutical material science paradigm provides strategies to address these requirements. Fundamentals of drug distribution modeling in the body via pharmacokinetics and dynamics as well as a highly encouraging case study on nanoparticle-based oral delivery are presented in Section 3. Our main focus will be on the process technologies for the formation of ONP by top–down and bottom–up approaches as discussed in Section 4. We highlight the relevance of material and process functions as key aspects in process and product design that lead to the desired product properties, as depicted in Figure 1. In particular, we introduce a predictive model for nanoparticle anti-solvent precipitation. We further shed light on the complex interaction of size reduction and ripening during bead milling and discuss promising options to model size reduction. Finally, Section 5 concludes the paper by pointing out areas for future developments, leveraging hybrid approaches combining first principles-based models with artificial intelligence.

## 2. Background

Nano-based solutions to improve drug solubility and bioavailability are a promising approach if industrial requirements for the drug delivery challenge are met, as will be pointed out in Section 2.1. It will become clear that the selection of nano-based solutions still is heuristics-based and highly empirical due to the underlying scientific complexity and to preference of the pharmaceutical industry for proven methods. The particle technology approach in this paper—predicting the properties of novel materials from first principles using advanced simulation techniques and modern computational techniques—is consistent with the pharmaceutical material science paradigm as laid out in Section 2.2. This has the advantages of being both quicker and cheaper than a trial-and-error experimentation process, and it also yields detailed structural and dynamical information that can provide a stringent test of theoretical models.

### 2.1. Poorly Soluble Drug Formulation Routes

**Figure 2:**
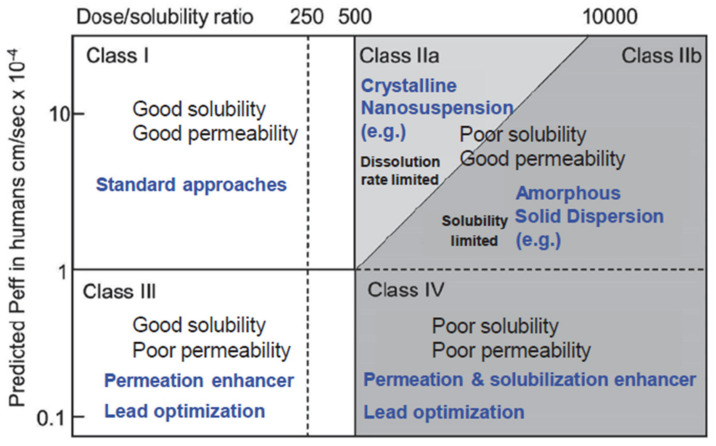
Modified biopharmaceutical classification system (BCS) including the division of class II in a dissolution rate limited partition for which nanotechnological approaches may be feasible (class IIa) and one that is solubility limited for which amorphous solid dispersions are most promising as drug delivery strategy (adapted with permission from [16], Springer Nature, 2015). Peff denotes average human jejunal permeability.

In drug delivery, there are various possible administration routes, but none is as popular and broadly accepted as the oral route owing to the multitude of advantages that are associated with it. To realize the efficient bioavailability of orally administered drugs, they must have enough aqueous solubility in order to get a therapeutic dose into the bloodstream of a patient. Unfortunately, due to the tendency of increasing complexity of the molecular structure of new drug compounds with their specific combination of hydrophobic and hydrophilic components and their location in the molecular structure, they often show neither global hydrophobic nor lipophilic properties and hence, they cannot be formulated with standard techniques. Thus, already, today, 40% of the top 200 oral drugs marketed in the US, 75% of compounds under development, and 90% of new chemical entities are classified as poorly soluble [14]. In the Biopharmaceutical Classification System (BCS), see Figure 2, many of these drugs are located either in class II, showing low solubility and high permeability through biologic membranes [15].

The limitations of amorphous solid dispersions (ASD) as the current standard drug formulation route for BCS II are related to the thermodynamic or kinetic stability of the amorphous single phase with the risk of drug phase separation and crystallization upon storage and/or limited solubility of the drug in pharmaceutically acceptable solvents with the need for huge solvent amounts during production routes. In addition, ASD are not applicable to drug forms for intravenous application. It is common to subdivide the BCS class II drugs into two subclasses: (a) Subclass IIa, which includes dissolution rate limited drug substances with high permeability and moderate solubility and (b) Subclass IIb, which includes solubility-limited drugs showing high permeability and low solubility [16,17]. Subclass IIa drugs are candidates for a nanocrystal formulation route if several industrial requirements are met.

Hence, more and more drug candidates are in the pipeline that cannot be treated with standard formulation technologies. Many companies are intensively investigating crystalline organic nanoparticles and their amorphous equivalents (ONP) process technologies that transfer the ONP benefits into tablets. To shorten the time for the formulation development and to make it more reliable, the goal is to early derive appropriate process parameters from distinct knowledge about material properties of the drug and excipients. In addition to “classical” laboratory-based work, also machine learning and other digital tools will increasingly accompany formulation development in order to achieve better and faster results for established but even more for novel formulation routes. Now novel formulation technologies such as nanotechnologies may be integrated into the standard formulation toolbox in the foreseeable future.

ONP are increasingly gaining interest as an alternative tool even though there are only few products on the market yet compared to ASDs [17]. Their advantage resides mainly in their high specific surface area and, only to a minor extent, in drug nanoparticle solubility increases. The drug nanoparticle solubility effect is small [18,19] even though the curvature of particle surfaces and the dissolution pressure increases with smaller particle size according to the Ostwald–Freundlich equation. For example, suppose the particle size is 150 nm. In that case, the solubility increases typically by just 15% in comparison to the bulk solubility. Consequently, particles must be considerably smaller than 100 nm, rather 10 nm, in order to obtain a substantially increased solubility that is comparable to that of ASDs [20]. As a positive side effect, it should be noted that the small differences in solubility between differently sized ONP in this size regime are responsible for only a little Ostwald-ripening with slow kinetics that is sometimes observed for nanosuspensions. Thus, stability against particle growth by Ostwald ripening can be mostly neglected.

The available specific surface area of the drug substance is increased by reducing particle size, thus improving the dissolution rate in a solvent. The relationship between the dissolution rate and the size of drug particles is described by the well-known Noyes–Whitney equation, which shows that the dissolution rater is proportional to the total surface area of the solid particles. The fast depletion of free drug molecules in the lumen is avoided, since a quick re-supply of them from the drug surface. An increase of the dissolution rate by a factor of 14 has been demonstrated for the drug Itraconazole when the particle size is reduced to 300 nm [21].

An additional benefit of nano-based formulations compared to their micro-sized counterparts is their strongly reduced food effect, meaning that the drug plasma concentration is much less dependent on food intake when the drug is administered orally and is therefore advantageous with respect to patient compliance [22]. The reduced food effect is well understood and can even be simulated with pharmacokinetic models [23]. The nature of the surface chemistry of the ONP is also important, which also influences their fate in the small intestine [24].

The production of ONP can be accomplished by bottom–up and top–down approaches. While top–down approaches involve comminution-based methods, bottom–up approaches are comprised of precipitation methods by adding anti-solvents to the drug solution. Crystalline and amorphous nanoparticles can be produced continuously by precipitation with a very small average size of even below 100 nm and narrow size distribution at the expense of low concentration of the obtained nanosuspension due to the limited solubility in pharmaceutically acceptable solvents. A large control over the particle size distribution for different solvents can be accomplished by the bottom–up approaches either in liquid [25] or in gaseous phase [26] and with a high throughput if secondary particle formation steps such as agglomeration and ripening are suppressed. The liquid phase must be removed to obtain a dry, fully re-dispersible powder for use in a solid dosage form such as a tablet. The numbering up of equipment is an elegant alternative to the challenging scale-up of precipitation technology.

An important exception is the production of Abraxane^®^ (Celgene, Summit, NJ, USA) with the anti-cancer drug paclitaxel, which is one of very few particulate drugs that is administered intravenously. However, it is not produced by a conventional anti-solvent precipitation route but via a special process technology (nanoparticle-albumin-bound™ (nab™) technology). In short, hereby, the drug is first dissolved in an organic solvent; then, it is emulsified in an aqueous phase that forms the continuous phase and contains human serum albumin (HSA) as a stabilizing agent. A following nanonization and high-pressure homogenization process comminutes the drug containing phase, which is then followed by a solvent extraction and drying process. By doing this, a re-dispersible powder with nanocrystals consisting of the drug and the stabilizer HSA only is obtained [27].

Top–down techniques such as wet media milling (WMM) and high-pressure homogenization (HPH) technologies are amenable to industrial production and are already applied for marketed products. While the HPH process relies on extreme shear forces and possibly cavitation, which are realized by pressing a suspension through gaps or slits and are applied to the drug crystals to disperse them [25], the process of WMM bases on forces that were generated by the impact of small ceramic balls onto the drug crystals. WWM is implemented by using planetary mills, but agitator bead mills are very common, because the underlying design can also be used for large-scale application, and the milling energy that acts on the material is much better controlled. Early development tests start with milling screening procedures to select those drug excipient combinations that ensure a stable suspension over a period of at least a few weeks. For this, planetary mills or other milling equipment is used that only needs tiny amounts of drugs of a few mg per trial, which is important due to its limited availability in the early stages [26,28]. Resonant acoustic mixing technology was further proposed as a promising variation of ball milling for which low-frequency acoustic waves are used for the size reduction of the drug particles in the suspension [29]. WMM was broadly introduced for drug comminution in 1991 by Sterling Winthrop [30]; since then, a huge number of papers discussing these technologies followed, and there are already some products on the market [31,32].

For more widespread uses, a nano-based technology track record has to be built by the community in addition to providing process understanding and ensuring scale up. The pharmaceutical industry will only apply well understood and scalable technology and continue to apply traditional formulation technologies as long as the dose of a specific drug for the needed therapeutic plasma level can be safely provided to the patient. More knowledge on the correct handling of nanoformulations along the entire process chain from the production of ONP suspensions to tableting of the dried powder on one hand and on the fate of the tableted drug in the gastro-intestinal (GI) tract from disintegrating of the tablet to absorption in the intestine on the other hand has to be built up to prevent dropouts in early formulation screenings. The physical stability of nanosuspensions against e.g., agglomeration in water and in biorelevant fluids (e.g., (e.g., fasted state simulated intestinal (FaSSIF) and gastric fluid (FaSSGF)) should be ensured to demonstrate the advantageous properties of nanocrystals in animal tests so that the drug can reach the absorbing intestine membranes of the test animal. The performance may be fine-tuned by adding further excipients such as ionic surfactants, disintegrants, or others. Very special attention must be paid to intravenous administration routes since the usable types of stabilizing excipients are very limited. Furthermore, not only agglomeration must be avoided but also the ONP must even dissolve very rapidly after injection to avoid any blockage in the bloodstream [33]. This was successfully achieved by EAGLE Pharmaceuticals, Inc. with their marketed drug Ryanodex^®^.

The physical stability of ONP should be preserved in each processing step throughout the whole process chain from drying, granulating, and mixing to tableting [34], even under GMP conditions. The desired ONP structure should be preserved throughout the process chain. Drying as the next step after milling means applying heat to the nanosuspension, which will also impact the compound quality. Drying with too low heat will lead to a compound with too high residual moisture load that has to be removed in an additional process step, whereas drying with too much heat will alter the product and respect re-dispersibility of the ONP embedded in the amorphous matrix [35]. Therefore, it is evident that each process step could lead to adverse effects such as the formation of mixed morphologies, e.g., crystalline parts consisting of mixed polymorphs or crystalline paired with amorphous proportions, which again lead to an undefined material with low reproducibility. In most cases, the industrial use of pharmaceutical forms requires morphology in pure form. This means that they must be either completely crystalline or amorphous but not a mixture of both, as the latter is not considered reliable for storage and is difficult to reproduce. Therefore, maintaining well-defined drug morphology is essential not only during production but also beyond, as the drug product must be storable for at least 3 years.

Regarding quality tests of a drug product, special attention must be paid to the required dissolution tests that measure the dissolution kinetics. Here, the separation of dissolved from undissolved drug in in vitro dissolution testing with conventional paddle tests according to the United States Pharmacopeia (USP 2) is crucial. In case the pore size of applied filters is not selected properly, undissolved ONP may pass. Filters with 20 nm pore size should be used in order to achieve good separation of undissolved particles as dissolved molecules and to avoid overestimating the performance of nanoformulations.

### 2.2. Particle Technology Applied to Drug Formulations

The application of particle technology concepts such as process and material models to the modeling and simulation of nanotechnology-based drug formulations in consistent with the pharmaceutical material science paradigm in pharmaceutical technology and the product design and engineering paradigm in chemical engineering. The combination of process and material models in a formulation process leads to the desired property function. Property functions of particulate products (property–structure functions) describe the desired property in dependence of the disperse properties, i.e., particle size, shape, structure, surface, and composition and their respective distributions. (Equation (1)):

property = f (particle size, shape, structure, surface, composition)(1)

In general, property functions are related to properties of the final product during application and to technological aspects, which include particle formation, formulation, and handling. With respect to application in pharmaceutical science and technology, property functions describe the target function, here bioavailability (see also Figure 1) and pharmacological efficacy, e.g., via solubility as function of particle size. In addition, aspects of particle formation, powder handling, for instance with respect to powder flow, or tableting in continuous production must be considered in any comprehensive approach.

The process function (process–structure functions) relates the process parameters to the product property (Equation (2)):

dispersity = g (process parameters, educt concentrations)(2)

Process parameters are the type of unit operations, their interconnection in the process, the process conditions under which the unit operations are operated (e.g., temperature, pressure, mass flow rates, etc.) and the materials that are processed. Structure–property as well as process–structure functions must be known in order to design optimal process variables and to achieve the desired goal, i.e., to produce well-defined, often multifunctional product properties. Usually, process chains (with or without recirculation loops) are employed during which both handling and end-use properties have to be optimized. The design of unit operations such as grinding, precipitation, granulation, or tableting strongly depends on material properties. These are best summarized in a general sense by a material function, which describes the influence of material properties on the performance of the respective unit operations. Examples will be discussed below for the two exemplary cases of grinding and precipitation.

Pharmaceutical Materials Science has been defined as follows [36,37,38]: The essence of pharmaceutical materials science is the application of fundamental concepts in the physical sciences to the challenges of understanding the behavior of soft, mostly organic, crystalline, and amorphous materials of relevance to the pharmaceutical industry. With modern computational techniques, it is now possible to predict the properties of novel materials from first principles using advanced simulation techniques. A truly holistic strategy for drug product development should focus on connecting solid form selection, particle engineering, and formulation design to both exploit opportunities to access simpler manufacturing operations and prevent failures [39].

**Figure 3:**
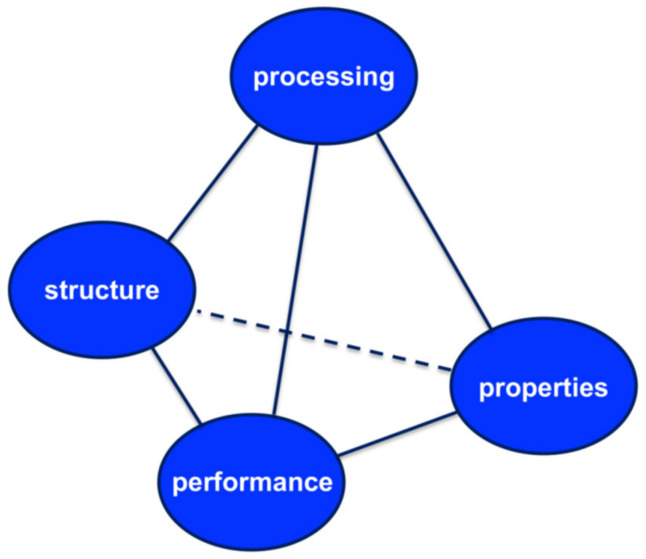
The pharmaceutical materials science tetrahedron. Reproduced with permission from [40], Elsevier, 2009.

The concept of materials science tetrahedron (MST, see Figure 3) concisely depicts the inter-dependent relationship among the structure, properties, performance, and processing of a drug [40]. It is proposed that a systematic implementation of MST can expedite the transformation of pharmaceutical product development from an art to a science. By following the principle of MST, an integration of research among different laboratories can be attained. The pharmaceutical science community can conduct more efficient, collaborative, and coherent research. Performance is determined by properties of the material that are in turn determined by its structure. In fact, the understanding the structure–property relationship is at the heart of materials science and process engineering. Once the relationship is clear, material properties can be modified by changing the structure of the matter through process engineering approaches and thereby delivering the desired performance.

Understanding the properties and behavior of pharmaceutical materials is critical to the design of a safe and effective dosage form [36]. In the future, product-process modeling and optimization will increasingly contribute to pharmaceutical product-process development [37]. First-principles and data-driven modeling approaches complement each other in pharmaceutical product-process development, for example for property prediction or for the formulation of a dynamic process model. However, a systematic framework is needed to work efficiently with product-process models and to fully exploit their potential benefits.

The concept of Pharmaceutical Materials Science in the pharmaceutical community has strong inherent similarities to the concept of product design and engineering (PDE) in the chemical engineering community. PDE is concerned with the definition of new and/or improved products based on the inputs of customer needs and/or new technologies [38,41]. PDE is the chemical engineering contribution to the new product development (NPD) workflow in the industrial sector such as the pharmaceutical industry. The fundamental aspects of product design and engineering have been described based on the following key terms: (i) the chemical product pyramid, (ii) a multi-faceted multiscale approach (nano, micro, meso, macro, mega scale), (iii) product and process design integration, all supporting (iv) chemical product design. The multifaceted multiscale approach enables the integrated view from the nanoscopic (molecular) end-use property up to the macroscopic (plant) level [42,43,44]. It is the beauty of the PDE concept that industrialization and manufacturing are explicitly addressed; the discovery does not stop at the lab or bench scale.

## 3. Modeling Particle Size-Dependent Dissolution and Absorption

The rate and extent of oral drug absorption in vivo are two key properties that decide the success of a drug development candidate. It is well known that a number of factors influence drug absorption from the GI tract after administration as a solid oral dosage form. The complex interplay between the events of drug release, dissolution, permeation across the intestinal epithelium, and pre-systemic metabolism in the gut wall and liver ultimately determines the rate and extent of systemic availability. In the pharmaceutical industry, dissolution testing and physiologically based absorption modeling are widely used to study this complex interplay.

Standardized in vitro dissolution test methods have been established to characterize the rate and extent of the drug release and dissolution from oral solid dosage forms. In combination with biorelevant dissolution media such as fasted (FaSSIF) or fed state simulated intestinal fluid (FeSSIF), these tests can be used to predict the in vivo dissolution behavior of orally administered dosage forms. The quantitative relationship between in vitro dissolution data and in vivo pharmacokinetic data is often referred to as “in vitro–in vivo correlation” (IVIVC). Several physiologically based models for GI transit and absorption have been developed. These aim toward a prediction of the in vivo oral drug absorption from a combination of a set of physiological properties such as dimensions and transit times of the GI tract as well as a set of physicochemical parameters/in vitro properties of the substance. Some of these models have become available in the form of commercial software tools such as GastroPlusTM and open-source initiatives such as PK-Sim^®^ [45], which is developed as part of the open systems pharmacology community [46].

The trigger for the current study was previous work [23] on the development of a mechanistic model that simulates the dissolution of a solid dosage form during GI transit under physiological conditions. For the evaluation of the model, cilostazol, a BCS class II (low solubility—high permeability) synthetic platelet inhibitor, was chosen because the dissolution and absorption behavior of this drug has been intensively studied [47] in vitro and in vivo. The authors measured the plasma kinetics of cilostazol after the administration of three different suspensions containing cilostazol with varying particle size distributions under fasted and fed conditions in beagle dogs. In addition, the in vitro dissolution profiles of the three types of suspensions were reported in both water and biorelevant dissolution media. Although the in vitro dissolution profiles showed an influence of particle size, the data were not able to quantitatively predict either the increase in bioavailability with decreasing particle size or the food effect observed in vivo. The aim of the previous work [23] was to demonstrate that the gap between the in vitro dissolution tests and the in vivo PK behavior can efficiently be bridged with the help of mechanistic, physiologically based pharmacokinetic simulations.

A previously developed physiologically-based pharmacokinetic (PBPK) model for gastro-intestinal transit and absorption was combined with a mechanistic dissolution model of the Noyes–Whitney type for spherical particles with a predefined particle size distribution [42]. In the model, the particles are grouped into *k* particle size groups. The number of particles in each group ($N_{i}$) is determined by the respective distribution function and remains constant over time. The initial amount of solid drug in each particle size group ($X_{o,i}$) is given by (Equation (3)):

(3) $$X_{o,i}=N_{i}\rho \frac{4}{3}\pi r_{o,i}^{3}\;\;\;\;\;\;\;\;i\in \left[1,\ldots ,k\right]$$

[43], where r denotes the density of the drug material, and r denotes the initial radius of the *i*-th particle size group. The sum of all initial drug amounts equals the total administered drug mass ($X_{o}$) (Equation (4)):

(4) $$\overset{k}{\underset{i=1}{\sum }}X_{o,i}=X_{o}$$

The dissolution process is described by a differential equation of the Noyes–Whitney type. The kinetics of the amount of solid ($X_{solid,i}$) and dissolved ($X_{dissolved,i}$) drug material are given by (Equation (5)):

(5) $$\frac{\partial X_{solid,i}}{\partial t}=-\zeta_{i}X_{o,i}^{1/3}X_{solid,i}^{2/3}\left(S_{int}-C_{lumen}\right)$$

and Equation (6)

(6) $$\frac{\partial X_{dissolved,i}}{\partial t}=-\zeta_{i}X_{o,i}^{\frac{1}{3}}X_{solid,i}^{\frac{2}{3}}\left(S_{int}-C_{lumen}\right)\;\;\;\;\;\;\;\;i\in \left[1,\ldots ,k\right]$$

with Equation (7):

(7) $$\zeta_{i}=\frac{3\;D}{\rho \;h\;r_{0,i}}\;\;\;\;\;\;\;i\in \left[1,\ldots ,k\right]$$

as derived in [44].

In these equations, $\zeta_{i}$ denotes a dissolution parameter that is constant for a given group of particles with radius $r_{0,i}$, D is the aqueous diffusion coefficient of the drug, and h is the thickness of the unstirred water layer. $S_{int}$ is the solubility of the drug in the intestinal fluid, and *C_lumen_* is the luminal concentration of the dissolved drug. *C_lumen_* is a function of time and the spatial coordinate in the intestinal tract and the solubility, which in turn can vary with the local pH in the intestinal lumen. This concentration is the driving force for passive diffusion across the intestinal epithelium and, consequently, *C_lumen_* is also dependent on the intestinal permeability, because absorption reduces the amount (and thus the concentration) of the drug in the lumen.

**Figure 4:**
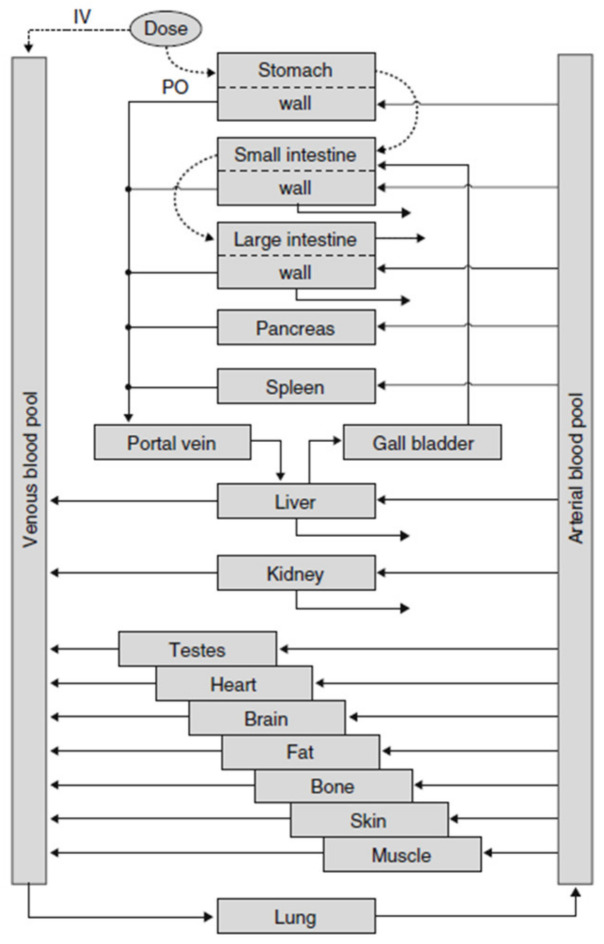
Structure of the whole PK-Sim^®^ simulation model with all organs (reprinted with permission from [48], Springer Nature, 2008). IV = intravenous, PO = oral.

In PK-Sim^®^ [45], the description of oral ADME is fully integrated into one simulation model, resulting in a complex model structure with the advantage that all processes can be described realistically and ensuring comparability of simulation results with pharmacokinetic experiments (see Figure 4). In PK-Sim, oral absorption is simulated as a “plug flow with dispersion model” which incorporates the small intestine as a single continuous compartment with spatially varying properties. The passage of a substance is described by a feeding-state-dependent, gastric-release function for the entrance into the gut and a transit function describing the transfer of the substance-containing package through the gut. At each point in time, the amount of substance absorbed into the portal vein is calculated. For solid formulations, the release of the substance into solution can be described according to predefined release function e.g., Noyes–Whitney type dissolution kinetics. For realistic simulation of the fate of the substance in subsequent ADME steps, the various organs are represented with blood flow rates, cross membrane permeation into organ tissue, and well as saturable metabolization processes.

**Figure 5:**
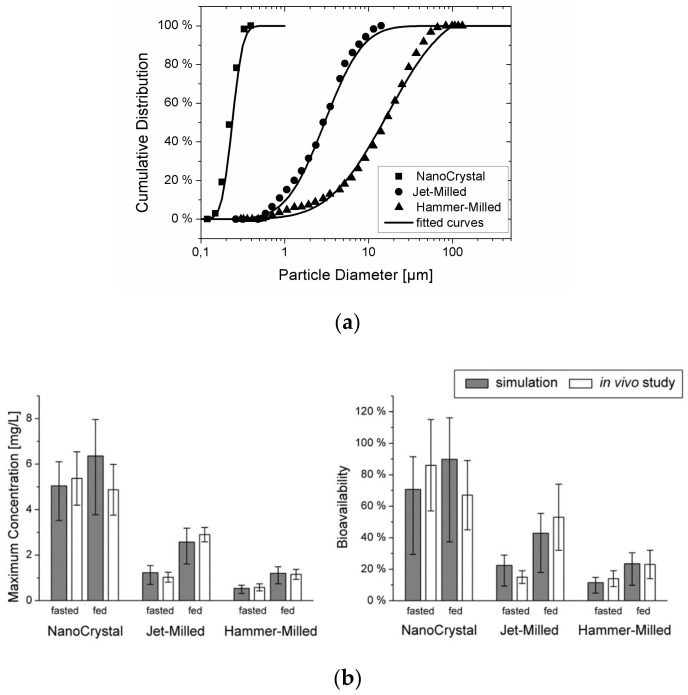
(**a**) Particle size distribution for the three cilostazol suspensions. Symbols represent the data from [47] (reprinted with permission from [23], Elsevier, 2010), the lines show the fit to a log-normal distribution function. (**b**) Comparison of the maximum concentration and bioavailability predicted from the particle size with the experimentally obtained values (mean and s.d. reprinted with permission from [23], Elsevier, 2010) of the three suspensions under fasted and fed conditions. The error bars represent the variability due to the inter-individual variability of the cilostazol clearance.

To validate the combined model, the plasma concentration–time curves for cilostazol obtained in beagle dogs using three different types of suspensions with varying particle diameters were simulated (see particle size data in Figure 5a). In vitro dissolution information was also available for the different formulations, but these data could only predict the in vivo outcome qualitatively. The mechanistic PBPK model could predict the influence of the particle size on the rate and extent of absorption under both fasted and fed conditions accurately, and the gap between the in vitro dissolution data and the in vivo outcome could successfully be explained (see Figure 5b). It was concluded that by integrating the processes of particle dissolution, gastro-intestinal transit, and permeation across the intestinal epithelium into a mechanistic model, oral drug absorption from suspensions can be predicted quantitatively. The model can be applied readily to typical formulation development data packages to better understand the relative importance of dissolution and permeability and pave the way for successful formulation of solid dosage forms.

## 4. Process Chain for Particle Formation and Formulation

**Figure 6:**
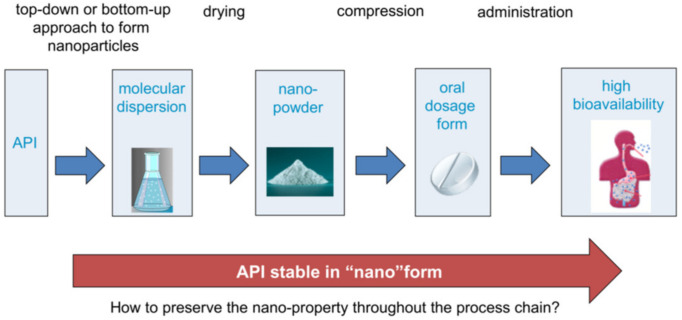
Exemplary process chain.

Within a collaboration between Bayer and the Erlangen cluster of Excellence “Engineering of Advanced Materials”, located at the university of Erlangen (FAU), five interconnected sub-projects studied the process chain from particle formation (see Figure 6) by top–down (nanomilling in a stirred media mill [49] and bottom–up nanoparticle formation by precipitation [50]. Particle formation was coupled to post-processing by formulation-supported spray drying and tablet formation [51] and comprehensive characterization along the process chain [52]. The latter included in situ techniques by small-angle X-ray (SAXS) and neuron scattering (SANS) to resolve nanoparticle formation even at short time scales and a wide range of methods for nanoparticle material characterization all the way up from formation to dissolution studies of formulated tablets.

### 4.1. Overview

The challenge of producing stable particulate dispersions in the smallest possible nanometer range can be addressed with two fundamentally different approaches, i.e., either by top–down or bottom–up methods as described in Section 2.1. Top–down approaches such as media milling can be operated at high particle concentrations while continuous operation and scale-up are possible. However, it is still difficult to reach particle sizes of a few 10 nm and narrow particle size distributions (PSDs). Furthermore, the risk of contaminating the product by the attrition of milling media must be carefully considered. One promising bottom–up approach is precipitation, which uses rather simple but continuous reactors. Prediction of the evolution of the particle size was demonstrated for organic compounds just recently; scale-up comes within reach and numbering-up by operating several reactors in parallel is another option for industrial production. However, the particle concentrations are so far rather low to avoid complications with particle stability and suspension rheology. In both cases, the formed nanoparticles must be stabilized sufficiently fast to prevent agglomeration and Ostwald ripening. In formulation technology, electrostatic, steric, or electrosteric stabilization is usually employed by applying polymeric additives, surfactants, or a combination of the two. Nevertheless, in most published papers and industry-oriented reports, the obtained particle sizes are larger than 100 nm. Furthermore, such stabilizers can cause severe alterations of the fluid flow due to changes in the viscosity and the dampening of turbulence and can impair the desired low solubility of the formed small particles by inducing complexation or ripening.

**Figure 7:**
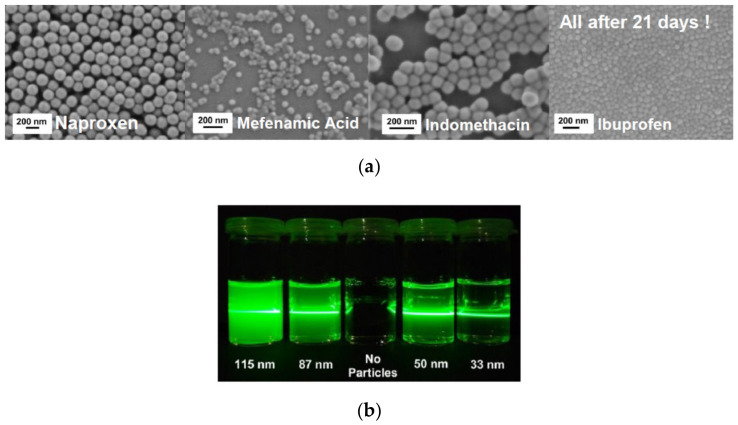
(**a**) Four different sub-100 nm, quasi spherical, and uniform amorphous organic drug nanoparticles (ONP) obtained by precipitation (SEM micrographs taken 3 weeks after production). (**b**) Tyndall effect to demonstrate the small particle size for Ibuprofen by reduced light scattering, particularly for the 33 nm particles.

Recently, we developed a mechanism based on multivalent metal cations, allowing stabilizing various hydroxyl groups containing drug nanoparticles (ONP) far below 100 nm [53] (the ONPs were stable for more than three weeks); see Figure 7. In this approach, no polymers or surfactants as stabilizing additives are needed, and hence no alterations of the fluid flow are expected. Therefore, the approach is ideally suited for comparison of experiments with precise simulations of the fluid mixing underlying the precipitation process. The ONP were produced by liquid anti-solvent and pH-shift precipitation utilizing in-house build static mixers. The influence of the mixing conditions on the final particle size distributions was studied by variation of the energy input during precipitation. Multivalent cations of a non-toxic metal can be used to achieve superior electrostatic stabilization of the precipitated ONP. For zirconium salts used as stabilizers in particular, the dependency of the resulting particle size on the pH and the salt concentration in the anti-solvent was investigated. Remarkably, our approach allows the continuous production of down to a few 10 nm in diameter. The amorphous character of the obtained particles was verified using X-ray diffraction and differential scanning calorimetry. To further demonstrate the broad applicability of our approach, the solvent was varied as well [53]. Remarkably, these particles are stable for at least several weeks. Currently, this approach is extended for bead milling as well. While electrostatic stabilization is rather well understood and predictable in the context of DLVO theory (named after Boris Derjaguin and Lev Landau, Evert Verwey and Theodoor Overbeek), steric stabilization is still largely developed empirically. Approaches driven by molecular simulations are still at a too early state and therefore mostly used to improve the understanding of interactions at model surfaces. In complex multi-component systems, which often are used in industry, any quantitative approach based on the prediction of particle interactions is not applicable. Artificial intelligence-based approaches in combination with high throughput and even automated characterization might offer solutions in future. Semi-empirical Hansen parameters are accessible and can used to classify the solubility of compounds according the well-known principle of similarity. Recently, the Hansen concept was adapted to the dispersibility of particles, which is accessible conveniently by sedimentation analysis [54]. These data are required for all methods for nanoparticle formation including anti-solvent precipitation and or nanogrinding, which are discussed in the following sections.

### 4.2. Precipitation

**Figure 8:**
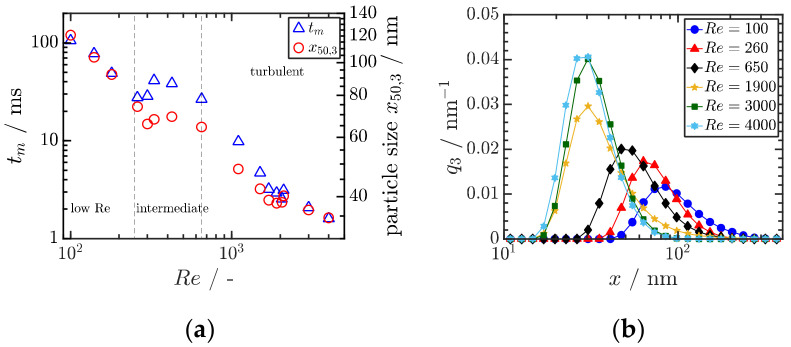
(**a**) Correlation between the experimentally determined mixing time $t_{m}$ and the mean particle as function of the Reynolds number Re. Reprinted with permission from [50], Wiley, 2019. (**b**) Measured particle size distributions in dependence of Re. Both results are obtained for Ibuprofen. Reprinted with permission from [50], Wiley, 2019.

The precipitation of amorphous and crystalline organic nanoparticles (ONP) is applied in various fields with a rising interest in the formulation of poorly soluble drugs. Key to the formation of ONP is the formation of a sufficiently high supersaturation as a thermodynamic driving force. Therefore, anti-solvent or pH-shift precipitation is employed for the production of ONP. Comprehensive combined experimental–computational studies in a simple T-shaped mixer for Reynolds numbers up to 4000 were conducted. In the experiments, micromixing times *t_m_* were determined for water–water and water–ethanol mixtures and compared to the measured mass median particle sizes *x*_50,3_ as shown in Figure 8a. The micromixing time is mainly determined by the power input as assumed in most mixing models. In particular, suitably manipulating the inflow conditions, the power input necessary to achieve a given micromixing time can be reduced by an order of magnitude [55]. Clearly, a higher Re number leads to smaller particle size due to enhanced mixing, which accelerates both nucleation and particle growth (Figure 8b). Particle sizes well below 100 nm can be achieved by proper stabilization against agglomeration and ripening.

In general, mass, momentum, and heat transfer processes coupled to chemical reactions produce nucleating species. Their distribution in the reactor related to their equilibrium concentration (or activity in the general sense) is defined as supersaturation *S*. It is the thermodynamic driving force for the phase transition and thus for the formation of a new particle phase. Depending on the spatial and temporal distribution of S in the reactor, nuclei form with a size distribution. Noteworthy, in the view of classical nucleation theory, the nucleation rate strongly depends on S. For instance, a high supersaturation with narrow distribution in time and space would lead to small nuclei with narrow PSD since all particles “experience a similar history”. After nucleation, several processes may occur sequentially or in parallel. These are growth processes for the further reduction of *S*, coagulation of the particles, their stabilization against coagulation, and eventually ripening effects in the liquid phase. This quite general framework forms the basis of any modeling approach for particle formation dynamics, which includes mixing, global reaction kinetics, nucleation, growth, agglomeration, and stabilization and even ripening.

The formation of ONP depends on the underlying phase diagrams and is controlled by chemical thermodynamics. However, equilibrium solubilities of even simple compounds are often unknown, in particular for complex molecules in pharmaceutical applications. A clear need exists to develop predictive methods for the determination of phase equilibria of particles. In other words, methods shall be developed to measure the material function of precipitation and crystallization, which then can be coupled to a process function as briefly discussed above.

Particle formation processes can further be subdivided in transport- and reaction-controlled processes. In reaction-controlled systems, e.g., in systems where mixing is much faster than the chemical reactions leading to precursor formation, the distribution of all reactions and thus of the supersaturation is much more uniform. Therefore, the formation of narrow PSDs will mostly occur in reaction-controlled systems. Firstly, particles form in a uniformly distributed nucleation burst, which is quickly reduced due to the formation of a new phase below the threshold value where homogeneous nucleation can occur. Secondly, the particles grow by the further reduction of the supersaturation until equilibrium is reached.

Typically, mass transfer issues are very common in liquid phase synthesis. The mixing intensity determines the local concentration fields and thus the supersaturation as driving force [56].

The energy dissipation in case of a stirred tank as well as in a continuous mixer such as a T- or Y-mixer is directly related to the volume-specific energy consumption. Higher energy dissipation will lead to successively smaller eddies in the fluid until the smallest eddy size (the Kolmogorov length scale) is reached. Mixing is shifted from macro- to meso-mixing and finally to diffusion-controlled micro-mixing [57,58]. The reactor design determines the residence time of particles and the local distribution of supersaturation. Both effects will control the width of the obtained PSD, or in other words, the width of the PSD is a measure of the mixing energy distribution. The full PSD can be modeled (at least for well-understood precipitation reactions) by a combination of direct numerical simulation (DNS) for complete resolution of the fluid flow coupled to an appropriate mixing model for mass transfer at the subgrid level on the one hand. The combination with a population balance model on the other hand [57,59,60,61] delivers the evolution of the particle size distribution.

The key aspects for predictive simulations are a detailed description of the spatiotemporal mixing process, sufficiently accurate data for equilibrium solubility, and a sufficiently large dataset to calibrate the nucleation kinetics by an estimation of the solid–liquid interfacial energy. In view of a quantitative agreement, recent findings suggest that in particular, the timescale ratio between the mixing process and solid formation, known as the Damköhler number, needs to be well captured in the simulations. In what follows, a fundamental concept to describe the anti-solvent precipitation on a macroscopic level is introduced, which allows predicting very well the trend of the median particle size as well as the entire shape of the particle size distribution at various process conditions for different mixing devices and solvent/anti-solvent pairs. The mixing process is described by the governing equations (in a Eulerian framework) for mass (Equation (8)):

(8) $$\partial_{t}\rho +\nabla \cdot \left(\rho u\right)=0$$

the volume fraction ϕ of the solvent/antisolvent pair (Equation (9)):

(9) $$\partial_{t}\rho \phi +\nabla \cdot \left(\rho \phi u\right)=\nabla \cdot D_{m}\nabla \phi$$

and momentum (Equation (10)):

(10) $$\partial_{t}\rho u+\nabla \cdot \left(\rho uu\right)=-\nabla p+\nabla \cdot \tau$$

where *u* is the velocity field, p is the pressure, and τ is the stress tensor including the viscosity [62]. It is important to note that the density (*ϕ*), the viscosity *μ*(*ϕ*), and the Diffusion coefficient *D_m_*(*ϕ*) depend on the volume fraction *ϕ* in case of a water–alcohol mixture [62]. The formation of the dispersed phase is governed by a population balance equation (PBE), Equation (11):

(11) $$\partial_{t}q\left(t,x_{p}\right)+\nabla \cdot \left(uq\right)+\partial_{x}\left(q\left(t,x_{p}\right)G\left[q\right]\left(t,x_{p},S\right)\right)=V\left(t,\phi \right)B_{hom}\left[q\right]\left(t,S\right)q_{c}\left(x_{p,c}\right)+B+D$$

where $q\left(t,x_{p}\right)$ and $q_{c}\left(x_{p,c}\right)$ is the number density of the dispersed phase $x_{p}$ and of the critical nucleus size $x_{p,c}$, respectively, $V\left(t\right)$ is the reaction volume, $B_{hom}$ is the homogeneous nucleation rate, G is the particle growth rate by diffusion or reaction, and B and D refer to birth and death terms due to aggregation, agglomeration, or ripening. The PBE is solved along Lagrangian trajectories, Equation (12)):

(12) $$\partial_{t}X_{i}=u$$

where $X_{i}$ is the spatial position in Lagrangian space. The coupling between the flow and the particle formation is accomplished by the mass balance $C_{l}=\phi C_{API,ini}-C_{S}$, whereby $C_{l}$ is the local concentration in the liquid, $C_{API,ini}$ is the initial concentration of the API, and $C_{S}$ is the solid concentration calculated as $C_{s}=\frac{\pi \rho_{p}}{6}\int_{0}^{\infty }x_{p}^{3}q\left(x_{p}\right)dx_{p}$ with the particle density $\rho_{p}$.

In the most general way, the evolution of the whole particle property space can be included by additional variables leading to multi-dimensional integro-differential equations [63]. Current research is directed toward the efficient coupling of PBE with computational fluid dynamics (CFD) simulations [61,63], to model increasingly complex reactions networks [64], to take several particle coordinates into account (e.g., size and shape), and to develop better kernels for agglomeration (e.g., complex fractal aggregates).

**Figure 9:**
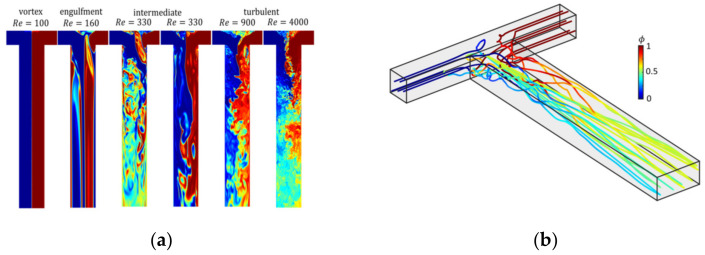
(**a**) Snapshots of the flow field in a T-mixer with increasing Re. Reproduced with permission from [55], Royal Society of Chemistry, 2019. (**b**) Lagrangian trajectories through the T-mixer, reprinted with permission from [50], Wiley, 2019.

Figure 9a shows the flow field obtained from assumption-free DNS simulation in a T-mixer at different Re-numbers up to 4000. The different flow regimes from laminar to intermediate and fully turbulent flow regimes are clearly depicted. Figure 9b shows trajectories of fluid parcels along which the population balance equation (Equation (11)) is solved. Along each trajectory, particle evolution is tracked, at the outlet of the mixer, the populations along each trajectory are mixed to compute the final particle size distribution.

The impact of fluid mixing on the precipitation of ONP is analyzed in depth by direct numerical simulations to determine the spatiotemporal evolution of the liquid phase composition and to estimate the particle evolution along Lagrangian trajectories. The revealed impact of mixing on precipitation enables a parameter-free estimation of the mean particle sizes and the particle size distributions. The distributions of residence time, supersaturation time, and particle size are self-similar in the turbulent regime and allow the derivation of scale-up rules.

**Figure 10:**
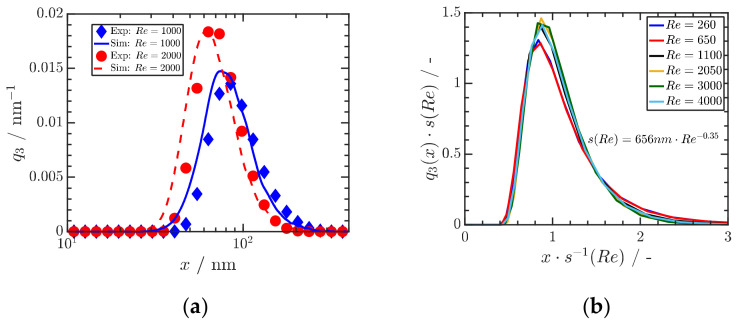
(**a**) Comparison of measured and calculated particle size distributions of Ibuprofen precipitated at three Reynolds numbers Re. An initial ibuprofen concentration of $C_{ibu}=30$ mg mL^−1^ is used, and ibuprofen is dissolved in ethanol. (**b**) Self-similar size distributions depend on Reynolds number Re. Reprinted with permission from [50], Wiley, 2019.

For the case of Ibuprofen for three different Reynolds numbers (Re) and thus mixing times, a quantitative comparison of experimental and numerical results is shown in Figure 10a. Figure 10b shows that the calculated particle size distributions are self-similar, providing a sound basis for scale-up. These remarkable results shows that (i) the precipitation of organic drug ONP in the range of a few 10 nm is possible by proper stabilization, (ii) that the obtained particle size distributions can be predicted by a knowledge-based quantification of mixing and particle formation and that (iii) the obtained size distributions are self-similar, which is the basis for scale-up to large scale [50].

### 4.3. Stirred Media Milling

**Figure 11:**
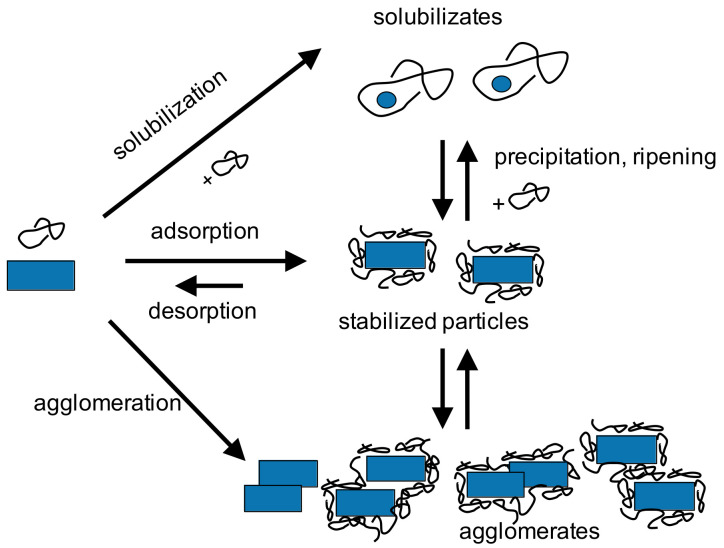
Complex interplay between fracture, surface activation, dissolution, and recrystallization, complex formation, and stabilization.

Particle formation by size reduction in stirred media mills allows the continuous and scalable production of particles below 1 μm and even nanocrystals. Process parameters (stirrer speed, temperature, bead size, and solvent) were systematically varied for various drug compounds and organic crystals. Grinding kinetics observed for batch and continuous operation are comparable under similar stressing and formulation conditions. Furthermore, it was found that the use of small grinding media, i.e., stress conditions where moderate stress energies but high stress numbers apply, are advantageous with respect to fast grinding kinetics and minimum energy consumption. Solubilization is an important factor that occurs of organic systems and easily can impair nanoparticle stability. Under such conditions, product characteristics are not only determined by pure breakage or colloidal stability but also by dissolution and ripening phenomena: Minimum product particle sizes at similar stressing conditions are observed under conditions where solubilization and ripening are minimized. Larger product particles are observed in systems with high solubilization capacities [65]. Then, the product particle size is rather determined by the (temperature- and solvent-dependent) solid–liquid equilibrium, i.e., dissolution and precipitation phenomena than by pure mechanical fracture. The complex interplay between fracture, surface activation, dissolution and recrystallization, complex formation, and stabilization is depicted in Figure 11.

**Figure 12:**
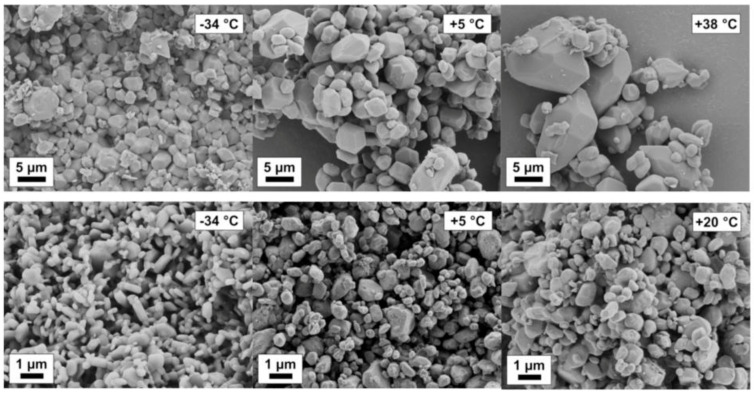
Naproxen particle ripening at varying temperatures in ethanol (**upper row**) and dichloromethane (**lower row**).

Mechanochemical effects can be particularly pronounced in organic systems. An increased solubility of stressed particles with respect to the equilibrium solubility of the solid has been observed. By means of NMR and RAMAN spectroscopy and thermodynamic considerations, solubility increases by chemical modification and isomerization of the solid can be detected. In fact, mechanical activation leads to an increase in solubility of the stressed solid, which was proven by solubility studies at different temperatures. The van’t Hoff enthalpy of dissolution of the stressed solid decreased remarkably in comparison to the enthalpy of the non-stressed solid as shown by the evaluation of van’t Hoff plots [49]. A positive effect of lowering the process temperature with respect to minimum product particle size was observed. Remarkably, the smallest product particle sizes were found for the lowest process temperature (251 K) at short process times (<30 min) and moderate stressing conditions; see Figure 12. In contrast, for the same stressing conditions at room temperature (293 K), much larger particles are obtained. Solvents will lower solubility lead to smaller particles, for instance, Naproxen in ethanol and dichloromethane.

Hence, the smallest product particle sizes were obtained using a polymeric stabilizer, which exhibits a high affinity to the model drug compound and a low solubilization capacity. A relationship between polymer affinity, solubilization capacity, and limiting product particle size has been observed, which supports the hypothesis that the final product particle sizes are rather determined by the solid–liquid equilibrium than by pure mechanical fracture [66].

The many different types of grinding machines for operation in the gas or liquid phase have in common that the design and the operational conditions determine the transport of the particles of the grinding zone (i.e., the process function). The transport depends on the mode of operation of the mill, i.e., flow rates, rotor speeds for instance, and the particles’ size, density, and concentration. The type of stressing can be one-sided as in impact mills or jet mills, or two-sided as in ball mills or roller mills operated in the gas phase or in bead mills operated in the liquid phase. Upon stressing, energy is transferred to the particles, which in turn deforms elastically and plastically. Only the elastically stored energy is available for fracture. Fracture typically occurs at internal defects in the crystal lattice of the particles. Once the elastically stored energy is larger than the energy required for crack opening, fracture occurs. In a meaningful simplification, grinding is characterized by just two variables, namely the stress energy (SE) per stress event and the number of stress events (SN) that a particle experiences in the mill [67]. Then, the supplied energy per mass of product $E_{m}$ is given by Equation (13):

(13) $$E_{m}∼SE\cdot SN$$

In principle, the variables SE and SN can be determined for any specific mill by proper simulation of the two-phase flow in the mill via CFD simulations. This approach is straightforward for sieve or classifier hammer mills operated in the gas phase at particle concentrations below a few 100 g/m^3^ [68]. The situation is more complex in (fluidized bed opposed) jet mills in the gas phase or in bead mills in the liquid phase. In the former case, high gas velocities are rather difficult to handle due to the compressibility of the gas; in the latter case, the filling ratio of the beads of roughly 80 vol-% and the high particle concentrations of several 10 vol-% induce a strong phase coupling between the two solid phases (beads and product particles) and the liquid phase. Therefore, CFD models must be coupled to discrete element method (DEM) simulations to account for the momentum transfer between the fluid and the particle phase at elevated concentrations and to determine SE and SN.

Recently, it was shown that the deformation of spherical probe particles can be used to directly measure the absorbed stress energy from their plastic deformation detected by image analysis. This approach is linked to SEM-based single particle stressing where measured stress–strain curves of compressed probe particles, for instance ductile metal particles, can be modeled by FEM simulation [69]. This combined experimental–theoretical approach provides a direct link to the reaction of the stressed particles under the influence of SE [70].

The reaction of a stressed particle can be condensed into a complex material function, which depends on several material parameters such as Young’s modulus, Poisson ratio, hardness, fracture toughness, or brittle–ductile transition. Since elastic and inelastic deformation as well as fracture strongly depend on the particle’s internal defect structure, multiscale approaches such as molecular simulations coupled to continuum fracture mechanics are of fundamental interest [71] but cannot predict the outcome of a fracturing event due to the largely unknown defect structures. However, the outcome of fracture can be described by the breakage probability and the breakage function. Both depend on the absorbed energy, the particle size, and the intrinsic material parameters.

**Figure 13:**
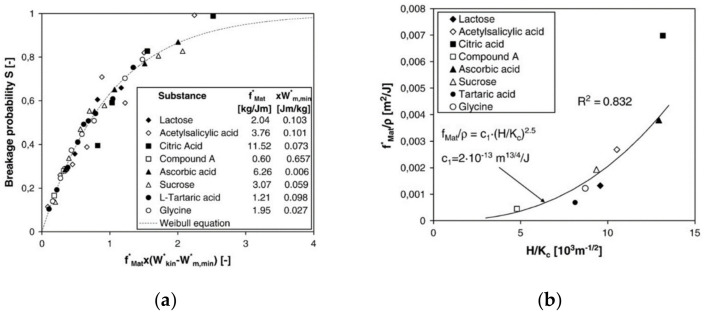
(**a**) Master curve for the breakage probability S by impaction (reprinted with permission from [72], Elsevier, 2009). (**b**) Material parameter $f_{mat}^{*}$ divided by the particle density as function of hardness H and fracture toughness $K_{c}$ (reprinted with permission from [72], Elsevier, 2009).

The breakage probability S (Equation (14)) of many different types of particles including organic crystals has been modeled for the one-sided impaction of particles by a unique master curve [72], see Figure 13a:

(14) $$S=1-\exp\left(-f_{Mat}^{*}{\left(1+\frac{E}{E_{target}}\right)}^{-1}kx\left(W_{kin}-\left(1+\frac{E}{E_{target}}\right)W_{m,min}^{*}\right)\right)$$

For soft particles impacting on hard targets, the relative effects of the Young’s moduli of particle E and target $E_{target}$ can be neglected. The breakage function can be described by the superposition of at least two lognormal distributions. The mean sizes, the standard deviations of the two sub-distributions, and the coupling parameter between them all depend on the parameters $f_{Mat}^{*}$ and $W_{m,min}^{*}$ in (Equation (14)) [73]. Interestingly, both parameters can be modeled as a function of fracture toughness K_C_ and hardness H, i.e., in dependence of the intrinsic material parameters (see Figure 13b). In principle, these parameters can be determined by nanoindentation; however, this approach is rather tedious due to the high number of measurements on single particles for statistically reliable data. More straightforward is the direct measurements of $f_{Mat}^{*}$ and $W_{m,min}^{*}$ in single particle stressing events in model mills. Taking these different approaches together, the grinding behavior of hammer mills can be modeled very well [68].

### 4.4. Post-Processing and Modeling of Process Chains

**Figure 14:**
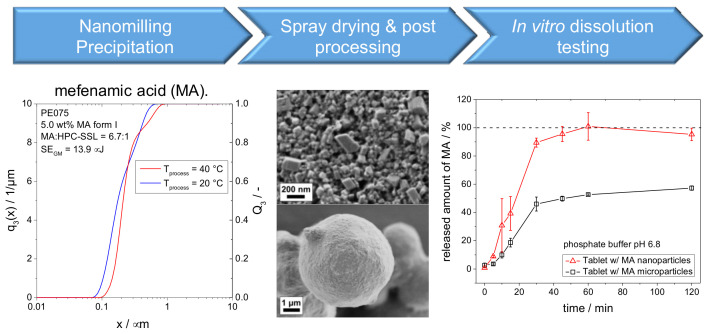
Process chain from nanoparticle formation via spray drying and post processing to in vitro dissolution testing.

An industrially feasible formulation approach combining media milling (or precipitation) and spray-drying was applied to improve dissolution characteristics of the poorly soluble drug mefenamic acid (MA), for instance (see Figure 14). The approach was studied for two MA polymorphs at different stressing and pH conditions. It was found that the final MA product particle sizes are rather determined by the solid–liquid equilibrium than by mechanical fracture. Obtained drug particles are only composed of the most stable polymorph. Direct compressed tablets containing MA nanocrystals exhibit a significant improvement of in vitro dissolution kinetics as compared to tablets with micronized drug particles [74].

The modeling of process chains with or without recirculation requires approaches that are known in chemical engineering as flowsheet simulations. These are state-of-the-art in fluids processing and are firmly based on phase equilibria and reaction rates of fluids. Classical approaches on product design were mostly built on these concepts, while distributed particle systems were widely neglected. The reasons can be seen in the difficulties to handle distributed properties, to model unit operations of particle technology such as size reduction, granulation, or tableting, and in the lack of available and reliable material functions. The modeling and simulation of particle formation and formulation must deal with highly complex and often transient two-phase flows and widely distributed particle phases in turbulent flows. On the one hand, time scales for particle formation can be very short in the order of milliseconds, while on the other hand, long-term stability must be guaranteed over months, as in pharmaceutical applications. Despite impressive progress in a few unit operations such as precipitation as shown above or fluidized bed granulation for instance [75,76], comprehensive approaches to handle other unit operations or their interconnection in complex processes are too often still missing.

A recent book on Dynamic Flowsheet Simulation of Solids Processes [77] is based on a six-years national German program of more than 20 groups and presents the latest advances in flowsheet simulation of solids processes, focusing on the dynamic behavior of systems with interconnected solids processing unit systems but also covering stationary simulation. The book includes the modeling of unit operations for the production and handling solids, for example by comminution, precipitation, classification, and granulation. New approaches for the description of solids and their property distributions are included as well. The mathematical treatment of flowsheets with multivariate population balances is a particular focus [77].

## 5. Conclusions and Unmet Needs

The overall approach to combine rationally-based and mechanistically-based models to address the entire value chain, i.e., multiscale modeling of the particle processes including computational fluid dynamics (CFD), discrete element method (DEM), population balance modeling (PBE), flowsheet simulation, Noyes–Whitney), and physiologically based absorption modeling (e.g., PBPK) will remain a long-term goal. Further improvements of models for unit operations in particle formation and processing are steadily becoming available by continuously improving and applying CFD-DEM-PBE models and their combination (process function). While the tools are available and “just” need to be further improved, their multiscale implementation for the predictive design of unit operations strongly depends on available material parameters such as mechanical and thermodynamic properties in dependence of particle size and shape (material function). The systematic characterization of particle properties combined with model-informed approaches to extract material data from model experiments is required to feed the available model “infrastructure”. High-throughput measurements and automated approaches might help in the future to reduce time and costs.

Predictive approaches are already available for well-defined systems with few components involved. However, their predictive power is so far limited to a few systems such as the precipitation of stabilized ONP in continuous T-mixers or hammer mills, for instance. One potential approach to overcome those limitations in data availability while simultaneously utilizing the established mechanistic insights in the future are so-called hybrid modeling approaches [78]. This might be realized in data-driven models combined with additional mechanistic input, e.g., meaningful chemical descriptors originating from quantum mechanical simulations or by directly coupling neural networks with mechanistic equations [79]. An AI-based evaluation of data may be applied to tackle complex issues of multi-component systems. The training of such systems can be based on data from all sorts of test results and even production plants. We envision that such approaches may lead to material property libraries. Once these are established, they are filled and continuously improved over time. These libraries may also contribute to the empirical or molecular property-based relations between the molecular structure and particle properties. For instance, a priori predictions of solubility only from molecular properties are currently beyond reach. Similar restrictions exist for the selection of molecular components for steric or electrosteric particle stabilization.

Even though the results of machine learning or artificial intelligence-based algorithms are promising, a key gap for a widespread usage seems to be having the data available to inform such a model. Different formulations, or even different manufacturing processes for the same formulation, often need completely separate descriptions, and each of them has a high dimensional space of potential influence factors including the active ingredient itself, often multiple excipients and manufacturing parameters that all would have to be characterized. Inherently, increased amounts of data are necessary to describe such a system with a sufficient generalization for future applications. This can be mitigated by standardized screenings [29,80] with a high throughput or robotic laboratory automation of such formulation assays. In those cases, established data-driven models can reduce the future experimental effort for new active pharmaceutical ingredients (API). This challenge is even more pronounced for new or not regularly utilized formulations. An argument for establishing standardized assays as early as possible for formulations is that many data-driven approaches also allow gaining at least rudimentary insights, such as the driving features for the formulation performance. This might give valuable insights in the further development.

**Figure 15:**
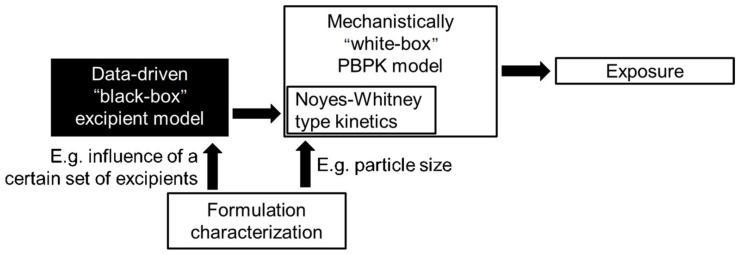
Exemplary model structure to integrate unknown excipients influence to the dissolution profile.

When finally trying to bridge the gap from formulation in vitro performance to in vivo performance, we have to keep in mind that in this regard, in vivo measures also have an inherent bias: naturally, only the formulations that showed the best in vitro performance enter in vivo trials. This makes it hard for a data-driven model to learn from potentially poor in vivo performances. The gap can be partially addressed by additional mechanistic modeling, e.g., by physiologically based absorption models, as shown in Section 2.2, which in turn can be coupled with data-driven models in a hybrid fashion to estimate the necessary inputs from the chemical structure of the active pharmaceutical ingredients and the utilized formulation. Figure 15 shows exemplary how, given sufficient data to inform the black-box part, one might include potential excipients for the nanoformulation whose influence on the dissolution profile via the Noyes–Whitney type kinetics Equations (5) and (6) might be hard to characterize in a mechanistic way.

In this case, the data-driven output would modify the dissolution kinetics depending on the chosen excipient. This is also an example where the data-driven model part might be easier to be informed independently by a sufficient number of in vitro measurements of dissolution kinetics in FaSSIF and FeSSIF contrary to few in vivo studies with a sufficient number of different formulations.

In this overview, we presented the long-term vision to combine modeling of drug administration with predictive models for product and process design. Nanoparticle-based oral delivery has the potential to become a next-generation formulation technology for dissolution-rate limited biopharmaceutical classification system (BCS) class IIa molecules if the following requisites are met: (i) quantitative understanding of the bioavailability enhancement benefit versus established formulation technologies and a reliable track-record of successful case studies are available; (ii) efficient experimentation workflows with minimum amount of active ingredient and a high degree of digitalization via e.g., automation and computer-based experimentation planning are implemented; (iii) scalability of the nanoparticle-based oral delivery formulation technology from lab to manufacturing is ensured.

By considering the whole process chain from the production of pharmaceutical ONP and the prediction of their properties toward whole body pharmacology, we showed remarkable progress at various levels but also identified considerable gaps and further needs. Only continuous improvements at all levels together with step-changing breakthroughs in predictive models will us bring closer to the long-term goal in pharmaceutical technology, i.e., the rigorous model-based development of products and processes for optimized bioavailability of a certain drug component.

## Data Availability

The data presented in this study are available on request from the corresponding author.
